# Interlaminar Granger causality and alpha oscillations in a model of macaque cortex

**DOI:** 10.1186/1471-2202-12-S1-P208

**Published:** 2011-07-18

**Authors:** Cliff C Kerr, Jue Mo, Samuel Neymotin, Mingzhou Ding, William W Lytton

**Affiliations:** 1 Department of Physiology and Pharmacology, SUNY Downstate Medical Center, Brooklyn, NY 11203, USA; 2 Complex Systems Group, School of Physics, University of Sydney, NSW 2006, Australia; 3 Department of Biomedical Engineering, University of Florida, Gainesville, FL 32611, USA; 4 SUNY Downstate/NYU-Poly Joint Biomedical Engineering Program, Brooklyn, NY 11023, USA; 5 Kings County Hospital, Brooklyn, NY 11203, USA; 6 Department of Neurology, SUNY Downstate Medical Center, Brooklyn, NY 11203, USA

## 

A key part of accounting for the computational aspects of the brain is to describe the pattern of causality between spatially distinct regions. While there is evidence that feedforward and feedback mechanisms are both widespread, we hypothesize that causality in only one direction would typically predominate, especially in specialized circuits such as the sensory systems. This work uses both a cortical network model and *in vivo* experimental data from a visual attention task to investigate modulations in two related phenomena: alpha oscillations, and the strength and direction of causal interactions between cortical layers.

The cortical model consists of nine columns of six-layered cortex, comprising a total of 4230 event-driven, rule-based neurons distributed among 13 distinct cell populations [1]. Inter- and intra-columnar connections are based on known anatomical connectivity patterns. The model is driven by subthreshold, Poisson-distributed synaptic input; attentional effects are modeled as increased input to supragranular layers. Output from the model was compared to experimental data recorded from two macaques performing a sensory discrimination task [2,3]. Linear array multielectrodes were chronically implanted in inferotemporal cortex (IT), allowing 14-channel local field potentials (LFPs) and multiunit activities to be recorded from each cortical layer during attend and ignore conditions.

Experimentally, it was found that LFP alpha power was higher in the attend condition than the ignore condition, with a greater increase in supragranular layers (19% increase in power) than in infragranular ones (13% increase). The cortical model produced similar results: in the attention condition, the overall increase in alpha power was 18%, with a 27% increase in supragranular layers and a 10% increase in infragranular ones. In both the model and experiment, the strongest causal influence was from the supragranular layers to the infragranular ones; causal involvement of the granular layer was limited. Maximum causality from supragranular to infragranular layers occurred at frequencies in the alpha band, and was modulated by attention: experimentally, maximum causality was found at 10 Hz and 12 Hz in the ignore and attend conditions, respectively, while the model showed maximum causality at 8 Hz and 11 Hz for ignore and attend conditions, respectively.

In summary, we demonstrate a cortical network model that can reproduce several key features of experimental LFP data in IT, including that (1) alpha power increases with attention, (2) there is greater modulation of alpha in supragranular than infragranular layers, and (3) the predominant direction of causality is from supragranular to infragranular layers.

## References

[B1] Neymotin SA Jacobs KM Fenton AA Lytton WW Synaptic information transfer in computer models of neocortical columns J Comput Neurosci in press DOI:10.1007/s10827-010-0253-4 20556639 10.1007/s10827-010-0253-4 PMC2997390

[B2] Bollimunta A Chen Y Schroeder CE Ding M Neuronal mechanisms of cortical alpha oscillations in awake-behaving macaques J Neurosci 2008 28 9976 9988 18829955 10.1523/JNEUROSCI.2699-08.2008 PMC2692971

[B3] Mo J Schroeder CE Ding M Attentional Modulation of Alpha Oscillations in Macaque Inferotemporal Cortex J Neurosci 2011 31 878 882 10.1523/JNEUROSCI.5295-10.2011 21248111 PMC6632909

